# Myocardial Protection in Cardiac Surgery: Exploring the Influence of Anesthetic Agents

**DOI:** 10.5152/eurasianjmed.2023.23376

**Published:** 2023-12-01

**Authors:** Burhan Dost, Esra Turunc, Elif Sarikaya Ozel, Muhammed Enes Aydın, Yunus Emre Karapinar, Muzeyyen Beldagli, Alessandro De Cassai

**Affiliations:** 1 Department of Anaesthesiology and Reanimation, Ondokuz Mayıs University Faculty of Medicine, Samsun, Türkiye; 2 Department of Anesthesiology and Reanimation, Atatürk University Faculty of Medicine, Erzurum, Türkiye; 3 Department of Anesthesiology and Reanimation, Samsun Training and Research Hospital Faculty of Medicine, Samsun, Türkiye; 4 UOC Anesthesia and Intensive Care Unit “Sant’Antonio”, University Hospital of Padua, Padua, Italy

**Keywords:** Cardiac surgery, inhalation anesthetics, ischemia–reperfusion injury, myocardial protection, propofol, preconditioning

## Abstract

This review assesses the efficacy of inhalation anesthetics and propofol in cardiac surgery, primarily focusing on their impact on myocardial protection and subsequent clinical outcomes. The review provides a concise summary of the current scientific information regarding the protective effects of inhalation anesthetics and propofol, particularly in the context of ischemia–reperfusion injury during cardiac surgery. The review delves into the mechanisms of action and discusses clinical studies comparing the 2 anesthetic strategies regarding mortality, complication rates, and length of hospital stay.

Inhalation anesthetics exhibit cardioprotective properties through many mechanisms, such as preconditioning, scavenging of free radicals, and stabilizing mitochondria. Propofol demonstrates certain protective benefits but does not possess the preconditioning capability of inhalation medications. Clinical investigations yield contradictory findings, as several studies indicate enhanced outcomes with inhalation anesthetics, while others observe no substantial disparity between the 2 approaches. The cardioprotective efficacy of propofol against ischemia–reperfusion injury remains limited. While its inherent antioxidant properties offer direct myocardial protection, propofol demonstrably lacks the preconditioning-mediated signaling pathways triggered by inhalation anesthetics. As a result, propofol’s protective effect may be slightly inferior to preconditioning strategies, and its potential to inhibit organ-protective impact of other interventions needs further investigation.

The question of which anesthetic approach offers superior myocardial protection remains debatable. Current evidence is inconclusive, potentially due to patient heterogeneity, surgical complexity, and methodological limitations of existing studies. Future research, including pharmacogenetic studies and large, well-designed, randomized controlled trials, are necessary to provide definitive guidance on anesthetic selection for optimal myocardial protection in cardiac surgery.

**Main Points**

- Inhalation anesthetics demonstrate cardioprotective properties by preconditioning.
- Propofol, despite its quick onset, falls short in myocardial protection compared to inhalation anesthetics.
- Mortality studies comparing inhalation and total intravenous anesthesia (TIVA) in cardiac surgery are inconsistent.
- Comparative analyses of TIVA and inhalation anesthesia in valve surgeries reveal no significant differences, creating uncertainty in optimal cardiac surgery anesthetic.
- Inconclusive evidence highlights the need for multicenter trials and standardized protocols to determine the ideal cardiac surgical anesthetic.

## Introduction

Anesthetic management for cardiac surgery has improved significantly in the recent years. The traditional cardiac surgical procedure involving a median sternotomy has brought about various factors affecting anesthesia management. To blunt the acute sympathetic response and enhance hemodynamic stability, the historical approach has focused on the use of high-dose opioid regimens. However, this method is associated with drawbacks such as prolonged postoperative mechanical ventilation, extended stays in the intensive care unit and hospital, along with other well-documented side effects. Instead of the long-practiced technique of high-dose opioid-based intravenous anesthesia, the fast-track anesthesia approach, initiated in the 1990s, has shifted toward a balanced anesthesia application. With this new approach, anesthesia induction involves short-acting intravenous anesthetics such as propofol, etomidate, or midazolam, combined with low-dose opioids and moderate-acting neuromuscular blockers like rocuronium or cisatracurium. Maintenance is then sustained with intravenous agents or volatile anesthetics, along with low to moderate doses of opioids and neuromuscular blockers.^1^ Also, opioid-sparing techniques like regional anesthesia reduce opioid requirements and length of hospital stay and are associated with significantly improved perioperative outcomes.^2-4^

For years, inhalation anesthetics were primarily associated with undesirable effects such as suppression of myocardial contractility, vasodilation, and coronary steal. However, in recent years, the demonstration of their positive impact in preconditioning and postconditioning has made these agents appealing in cardiac surgery. In fact, the detailed guidelines on the perioperative use of inhalation anesthetics during bypass surgery were outlined by the European Association for Cardio-Thoracic Surgery in the perioperative medication guide for adult cardiac surgery published in 2019.^5^

Cardiac surgery induces prolonged global ischemia.^6,7^ At this point, our primary objective is to enhance the cell’s resistance to ischemia, thereby extending the myocardium’s tolerance to anaerobic metabolism. Recently, several organ-protective techniques have come to the forefront for this purpose. Preconditioning refers to a state where a temporary ischemic or stressful event is induced or mimicked to aid vital organs in enduring the subsequent prolonged ischemic event. There are different types of preconditioning, such as ischemic preconditioning, inhalation preconditioning, and remote ischemic preconditioning. These preconditioning methods make the myocardium more resilient to future ischemic events.^8^

The reperfusion stage, where blood flow is restored to the myocardium, can be described as a “paradoxical phenomenon.” While reperfusion may reduce the infarcted area, it can also lead to myocardial dysfunction, increased apoptosis, and further damage.^9,10^ During the reperfusion period, the rapid entry of molecular oxygen into the cell generates free oxygen radicals. These radicals are among the primary factors responsible for reperfusion injury. Cellular structures, especially membrane lipids, proteins, nucleic acids, and deoxyribonucleic acid molecules, change under the influence of these radicals. As a result, adverse effects such as myocardial stunning, reperfusion arrhythmias, necrosis in myocytes, and dysfunction of coronary endothelium and microvasculature can be observed. The release of cytokines due to inflammation causes cellular damage, and disruption in calcium transport occurs following damage to the sarcoplasmic reticulum during ischemia, leading to intracellular calcium increase. Simultaneously, the opening of the mitochondrial permeability transition pore (mPTP) during reperfusion allows for further calcium accumulation inside the cell. This situation leads to hypercontractility and reperfusion arrhythmias.^11,12^

**Figure 1.**
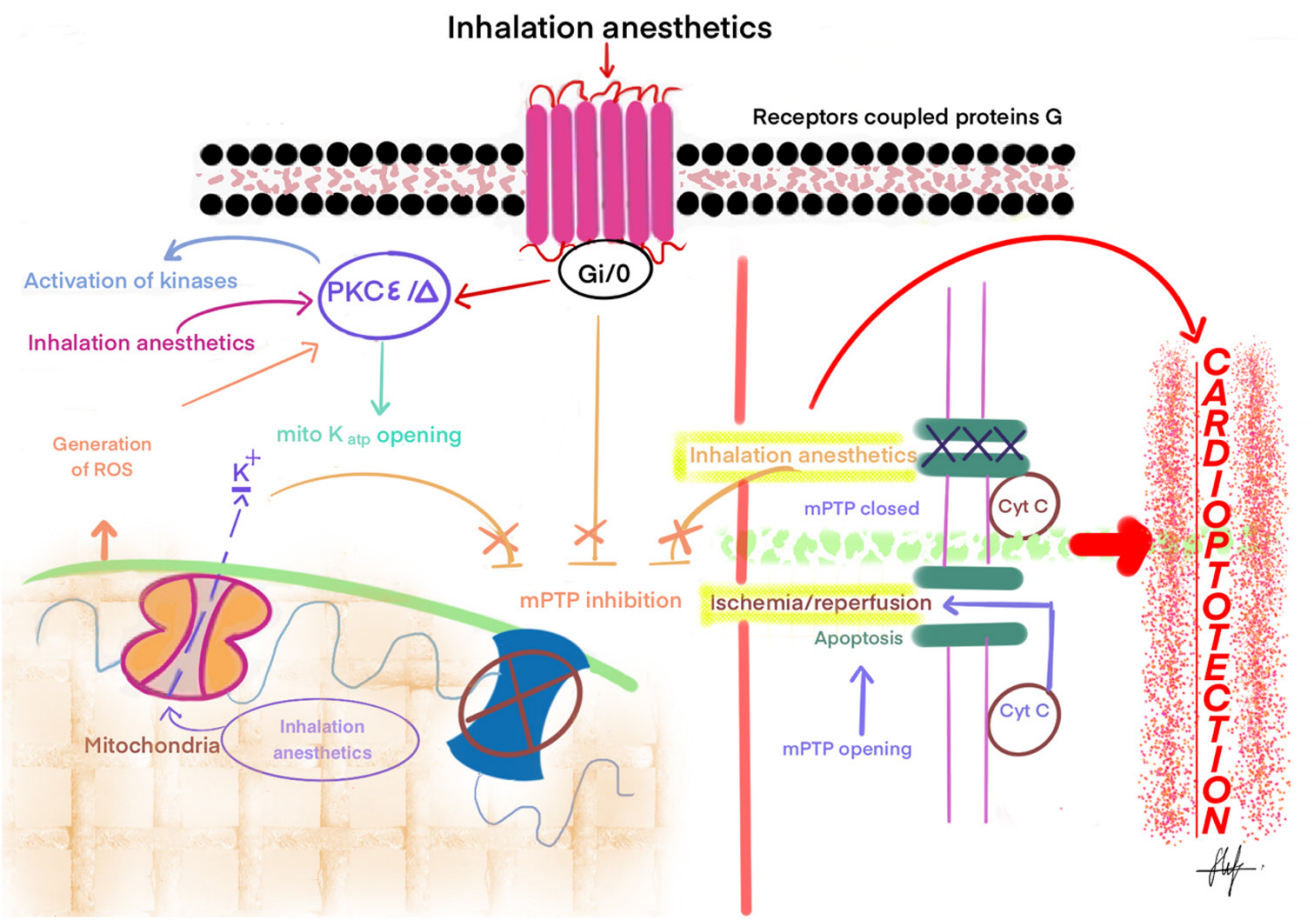
Mechanism of action of inhalation anesthetics on G protein-coupled receptors and cellular signaling pathways. The comprehensive overview shown here provides a visual representation of the intricate molecular and cellular events triggered by inhalation anesthetics, shedding light on their role in conditioning and the modulation of various cellular components. mPTP, mitochondrial permeability transition pore; PKCε, protein kinase C ε; ROS, reactive oxygen species.

Mitochondrial permeability transition pore was first characterized in reproductive cells by Haworth and Hunter in the 1970s. This complex is a structure that regulates membrane permeability in the inner mitochondrial membrane. However, reperfusion may be a crucial step in sustaining the optimum conditions for opening mPTP, potentially initiating cell death. The transient opening of mPTP has a physiological role related to the homeostasis of free oxygen radicals and calcium release. Therefore, it has been demonstrated that opening mPTP during ischemic pre-conditioning can provide cardioprotection. The accumulation of lactic acid during ischemia has a potent inhibitory effect on mPTP. However, the rapid breakdown of lactic acid and the reactivation of Na–HCO_3_ symporter by Na–H exchanger in the first few minutes of reperfusion allow the physiological pH level to be restored during reperfusion. This condition allows the opening of mPTP. However, prolonged opening of mPTP leads to increased permeability of the inner mitochondrial membrane to <1.5 kDa molecules, a decrease in membrane potential, disruption of oxidative phosphorylation, ATP consumption, and the release of pro-apoptotic factors. As a result, this situation can trigger cell death.^13,14^ In conditioning, the primary effects of inhalation anesthetics are observed to be through the modulation of G protein-coupled receptors on the cell surface. This effect leads to the activation of various protein kinases and the stimulation of cellular signaling pathways. Additionally, it controls the production of free oxygen radicals and initiates protective mechanisms through the activation of ATP-sensitive mitochondrial potassium channels and mPTP inhibition (Figure 1). The duration of the protective effects of anesthetic conditioning is crucial, typically remaining active for 1 to 2 hours. Two main pathways support this process: in RISK (reperfusion injury salvage kinase) pathway, cell surface receptors and G-protein-associated receptors initiate the protective pathway. Phosphatidylinositol (4,5)-bisphosphate 3-kinase (PI3K), protein kinase B, and other signaling pathways support cell survival and SAFE (survivor-activating factor enhancement) pathway. This pathway includes tumor necrosis factor alpha and signal transducer and transcription 3 (STAT-3) activator, mainly to reduce apoptotic cell death. Both paths have close interactions, and it is known that they convey protection through mPTP inhibition and activation of KATP channels. The second protection window emerges 24-48 hours later and lasts up to 72 hours. This involves increased expression of protective proteins in response to acute signaling, including protein kinase C ε, STAT, and nuclear factor-κB.^15-17^

Reperfusion injury can be attributed to the rapid influx of molecular oxygen into the cell during reperfusion, leading to the generation of free oxygen radicals. These radicals have the potential to impact cellular structures, causing damage to nucleic acids, proteins, and membrane lipids. Additionally, they can initiate the inflammatory process. The rapid clearance of accumulated lactic acid during reperfusion, which occurs during ischemia, can trigger the formation of free oxygen radicals. Therefore, controlling free oxygen radicals is crucial for protecting the myocardium.

In conclusion, inhalation anesthetics reduce the excessive production of free oxygen radicals post ischemia–reperfusion, thus alleviating the oxidative stress that triggers reperfusion injury. ATP-sensitive potassium channels are a direct target of inhalation anesthetics, regulating mitochondrial volume and homeostasis. They decrease the production of excessive free oxygen radicals and mitochondrial calcium accumulation, providing an optimal environment for ATP production and inhibiting mPTP.

### Clinical and Research Consequences

Propofol is an anesthetic agent used in approximately 100 million surgical procedures annually. Its rapid onset, short duration of action, antiemetic effects, and low side effect profile have made it highly popular in anesthesia practice.^18^ Additionally, it possesses antioxidant properties that may contribute to myocardial protection. It has been demonstrated to have the ability to protect the myocardium by reducing the production of excessive free oxygen radicals during ischemia–reperfusion injury.^19^ This is also a fundamental mechanism by which propofol provides protection against myocardial ischemia–reperfusion injury. Additionally, it ensures the stabilization of the mitochondrial membrane by reducing mitochondrial calcium uptake and directly inhibiting the mPTP. In contrast to inhalation anesthetics and opioids, it has been shown that propofol does not trigger signaling pathways associated with preconditioning, either before or after, and therefore does not exhibit any conditioning effect against myocardial ischemia–reperfusion injury. Overall, propofol’s protection against myocardial ischemia–reperfusion injury appears to be slightly less than the conditioning induced by inhalation anesthetics. It is reasonable to assume that propofol is not harmful by itself but likely inhibits organ-protective effects induced by other interventions.^20^

While the 2019 EACTS/EACTA/EBCP guidelines on cardiopulmonary bypass in adult cardiac surgery provide recommendations on the use of inhalation anesthetics in cardiopulmonary bypass,^5^ the 2021 ACC/AHA/SCAI Guideline highlights that, although the use of inhalation anesthetics in cardiac surgery allows for early extubation, no positive effect on mortality has been demonstrated.^21^ This conclusion is based on the ‘The Mortality in Cardiac Surgery Randomized Controlled Trial of Volatile Anesthetics (MYRIAD)’ study, representing the largest patient population in the literature comparing inhalation and intravenous anesthetics.^22^ This multicenter, prospective study aimed to compare total intravenous anesthesia (TIVA) with inhalation anesthetics in cardiac surgery and was planned to include 10,600 patients from 36 centers in 13 different countries. However, the initial results of this study were deemed insufficient due to the slight 1-year mortality difference of only 0.2% between the 2 groups, and it was terminated, citing the need for more patient data for statistical significance. Additionally, the fact that anesthesia management was left to anesthesiologists and inhalation agents were administered to only 478 patients in the inhalation anesthetics (2709) group during cardiopulmonary bypass raised concerns. In cases where high opioids were administered, the inhalation agent dose was often insufficient, and propofol, which could adversely affect the myocardial protective effects of inhalation anesthetics, was predominantly used, especially during induction. In the post hoc analysis of the study, myocardial infarction developing with hemodynamic instability within the first 48 hours postoperatively and cardiac-caused 1-year mortality was determined as primary outcomes. It was observed that the incidence of myocardial infarction with hemodynamic complications and cardiac-caused mortality was lower in the inhalation anesthesia group.

Following the MYRIAD study, additional research and meta-analyses have been conducted. For instance, Beverstock et al’s meta-analysis^23^ examined 40 randomized controlled trials comparing inhalation anesthesia with TIVA. No significant difference was found between the 2 groups in terms of 1-year mortality. However, a significant decrease in intensive care unit and hospital lengths of stay was observed in the inhalation anesthesia group. On the other hand, in another meta-analysis conducted by Bonanni et al,^24^ 42 randomized controlled trials involving 8197 patients were analyzed, evaluating mortality at 30 days and 1 year. There was no significant difference in short-term mortality between the groups, but the 1-year mortality was significantly lower in the inhalation anesthesia group (Inhalation: 5.5%, Propofol: 6.8%). An important distinction between these 2 meta-analyses is that Bonanni et al limited their analysis to studies that exclusively used cardiopulmonary bypass. In contrast, Beverstock et al’s meta-analysis had a broader scope, including patients undergoing off-pump coronary artery surgery. In a meta-analysis published in 2023 by Ren et al, the results of anesthesia management in valve surgeries showed no significant differences between TIVA and inhalation anesthesia in terms of mortality, myocardial infarction, arrhythmia, atrial fibrillation, pulmonary complications, neurological complications, and bleeding-related reoperations.^25^ In a meta-analysis published in 2023 focusing on the effects of propofol on survival, it was observed that propofol led to negative effects on mortality compared to other anesthesia agents. Particularly, it was concluded that it might reduce the chances of survival in perioperative and intensive care patients. However, the researchers emphasized the need for a careful risk-benefit analysis of propofol and suggested that large, pragmatic, multicenter randomized controlled trials are required for a definitive answer.^20^

## Conclusion

In conclusion, there appears to be insufficient information to determine a definitive preference between the 2 anesthesia methods. Most studies present conflicting results. Patient characteristics and the complexity of surgical procedures pose challenges in anesthesia selection, emphasizing the need for a careful patient assessment and consideration of surgical requirements. Additionally, the lack of precise results regarding myocardial damage markers raises some doubts about the effectiveness of damage. Therefore, the question of which anesthetic method is more effective in myocardial protection remains unclear. Future pharmacogenetic and pharmacogenomic studies and extensive, randomized, multicenter trials may provide clearer guidance on anesthesia selection.
